# One Health Perspective on Antimicrobial Resistance in Bovine Mastitis Pathogens—A Narrative Review

**DOI:** 10.3390/antibiotics15010084

**Published:** 2026-01-14

**Authors:** Bigya Dhital, Rameshwor Pudasaini, Jui-Chun Hsieh, Ramchandra Pudasaini, Ying-Tsong Chen, Day-Yu Chao, Hsin-I Chiang

**Affiliations:** 1 Department of Animal Science, National Chung Hsing University, Taichung 40227, Taiwan; 2 Department of Entomology, National Chung Hsing University, Taichung 40227, Taiwan; 3 Agricultural Research Development Program, Central State University, Wilberforce, OH 45384, USA; 4 Department of Veterinary Medicine, College of Veterinary Medicine, National Chung Hsing University, Taichung 40227, Taiwan; 5 Ministry of Agriculture and Livestock Development, Kathmandu 44600, Nepal; 6 Graduate Institute of Genomics and Bioinformatics, College of Life Sciences, National Chung Hsing University, Taichung 40227, Taiwan; 7 Graduate Institute of Microbiology and Public Health, College of Veterinary Medicine, National Chung Hsing University, Taichung 40227, Taiwan; 8 One Health Center, College of Veterinary Medicine, National Chung Hsing University, Taichung 40227, Taiwan

**Keywords:** One Health, public health, antimicrobial resistance, bovine mastitis

## Abstract

**Background/Objectives**: Bovine mastitis, a significant global concern in dairy farming, results in substantial economic losses and poses considerable risks to both animal and human health. With the increasing prevalence of antimicrobial resistance (AMR) in mastitis pathogens, the potential for resistant infections to spread from livestock to humans and the environment is becoming a critical public health issue. This narrative review summarizes the current evidence on antimicrobial resistance in pathogens causing bovine mastitis and examines it from a One Health perspective, encompassing animal, human, and environmental interfaces. **Results**: By examining the complex interplay among animal, human, and environmental health, we highlight key factors that drive resistance, including the overuse of antimicrobials, poor farm management, and environmental contamination. We also discuss innovative strategies, such as enhanced surveillance, pathogen-specific diagnostics, alternatives to antimicrobials, and sustainable farm practices, that can mitigate the emergence of resistance. Key knowledge gaps include a limited understanding of antimicrobial residues, resistant pathogens, and gene transmission pathways and inconsistent implementation of antimicrobial stewardship practices. **Conclusions**: This review emphasizes the need for a coordinated, multidisciplinary effort to reduce the burden of AMR in bovine mastitis pathogens, ensuring the continued efficacy of antimicrobials and safeguarding public health through responsible management and policy interventions.

## 1. Introduction

Bovine mastitis is the inflammation of the mammary glands and udder tissues due to microbial infections or physical injury. As one of the most common cattle diseases globally, it causes huge economic losses due to decreased milk production and low milk quality [1]. Among dairy diseases, mastitis is the greatest contributor to economic losses—about USD 65 billion per year globally, including USD 24.5 billion in Asia, USD 19.8 billion in Europe, USD 10.1 billion in North America, USD 4.6 billion in Latin America, USD 4.2 billion in Africa, and USD 1.6 billion in Oceania [2]. The economic losses due to mastitis are estimated at around USD 147 per cow per year and USD 146 billion worldwide [3,4]. A recent global analysis estimated that clinical mastitis and subclinical mastitis cause annual losses of about USD 13 billion and USD 9 billion, respectively [2]. Annual losses due to mastitis are estimated at around USD 2 billion in the USA, USD 1.6 to 2.2 billion in the EU, USD 310 million in Canada, USD 15–45 billion in China, and USD 2.2 billion in India [2,5,6,7]. Among total losses, mammary tissue damage leads to about a 70% reduction in milk production [8]. In addition to reduced milk yield and quality, medication and other expenses also contribute to the substantial losses attributable to mastitis in the dairy industry [9]. In severe cases, if not cured, mastitis becomes chronic or leads to reduced fertility or even death [4]. Bovine mastitis is classified into clinical, subclinical, and chronic forms based on the degree of inflammation. Clinical mastitis is easily detectable due to signs such as udder redness, heat, swelling, fever, dehydration, and changes in milk consistency, including flakes and clots [9]. It is further categorized into per-acute, acute, and sub-acute stages, depending on the severity [1,10]. In contrast, subclinical mastitis lacks visible symptoms, making it harder to detect, but is responsible for significantly higher economic losses and is more prevalent than clinical mastitis, with its incidence estimated to be 15–40 times higher [9,11]. Consequently, subclinical mastitis imposes a greater financial burden due to its increased prevalence and difficulty of detection [9]. Chronic mastitis causes persistent mammary gland inflammation for an extended period or recurs despite treatment, often requiring repeated antimicrobial therapy [12]. These repeated treatments create selective pressure that can drive the development and dissemination of antimicrobial-resistant pathogens.

Bovine mastitis, being one of the most prevalent diseases in dairy cattle [13], accounts for the majority of antimicrobial use in dairy farms, as treatment often involves repeated or empirical administration of antimicrobials [14]. This creates strong selection pressure that promotes the emergence and persistence of antimicrobial resistance (AMR) in pathogens. Subclinical and recurrent infections, as well as pathogen persistence in the udder and farm environment, further facilitate the development and dissemination of resistance [15]. Understanding this link within a One Health framework is crucial, as resistance genes can spread between humans, animals, and environments, highlighting the broader implications of mastitis management for antimicrobial resistance.

On-site antimicrobial treatment of bovine mastitis is commonly used to manage mastitis in dairy cows globally, with antimicrobials often administered before definitive diagnosis; however, in severe cases, systemic administration follows [16,17]. While this practice enables rapid intervention, repeated or empirically guided use exerts strong selective pressure, driving the emergence and persistence of AMR pathogens. Incomplete treatments or reliance on broad-spectrum antimicrobials further increases this risk, as resistant strains can persist in the udder, contaminate the farm environment, and potentially spread to humans and other animals [16,18].

According to the World Health Organization (WHO), “One Health is an integrated, unifying approach that aims to sustainably balance and optimize the health of people, animals and ecosystems” [19]. The One Health concept emphasizes the interconnections between human, animal, and environmental health, advocating for a unified approach to tackle global health challenges [10]. This strategy is jointly promoted by the WHO, the Food and Agriculture Organization (FAO), and the World Organization for Animal Health (WOAH). Originating as a response to the rising threat of zoonotic diseases, this framework has evolved to encompass broader issues, such as AMR, food safety, and environmental sustainability [20,21]. The concept is vital in tackling AMR, as resistant pathogens and antimicrobial residues can transfer between livestock, humans, and the environment, posing significant public health and ecological risks. By integrating expertise from veterinary medicine, human healthcare, agriculture, and environmental sciences, the One Health approach seeks to develop sustainable strategies to mitigate AMR while ensuring the health of all interconnected systems.

There is growing concern about the One Health perspective on AMR in livestock [20,22]. Given the extensive reliance on antimicrobials for the prevention and treatment of mastitis, it is crucial to explore and address AMR issues from a One Health perspective. Resistant bacteria, resistance genes, and antimicrobial residues can enter the environment through manure, wastewater, and agricultural runoff, contributing to broader ecological reservoirs of AMR. Similarly, resistant bacteria, resistance genes, and antimicrobial residues can indirectly enter humans through dairy or meat products. These pathways emphasize the importance of integrated surveillance and mitigation strategies that address AMR at the animal–human–environment interface. This narrative review discusses the implementation of One Health approaches to mitigate the spread of AMR and its associated risks to humans, livestock, and the environment.

## 2. Etiology of Bovine Mastitis

Bovine mastitis is associated with a combination of host, environmental, and management factors [23]. The disease typically shows a seasonal peak, with increased incidence during summer, when environmental conditions favour bacterial survival and transmission. For instance, one study showed that higher temperature and humidity during the summer favour bacterial growth, resulting in the highest risk of occurrence of clinical mastitis, with the highest risk in July [23]. Among lactation stages, early lactation is associated with higher susceptibility to mastitis due to the increased milk yield, immunosuppression, inadequate nutrition, and the heightened risk of udder injury during milking [24]. Increased milk yield and udder injury during milking can facilitate bacterial entry, while physiological stress during early lactation can lead to immunosuppression, reducing the cow’s ability to fight infections [25,26]. Similarly, inadequate nutrition can suppress immune function, further promoting infection [27].

Bovine mastitis can be caused by over 150 microorganisms, primarily bacteria, but also yeasts, viruses, algae, and fungi [28]. Key bacterial pathogens include *Staphylococcus* spp., *Streptococcus* spp., *Escherichia coli*, *Mycoplasma bovis*, and *Corynebacterium bovis* [28,29]. Pathogens are categorized as environmental or contagious. Environmental pathogens, such as *S. uberis*, *S. dysgalactiae*, coliforms, and *Arcanobacterium pyogenes* (now reclassified as *Trueperella pyogenes*), originate from the cow’s environment [9,30]. Contagious pathogens, including *S. agalactiae*, *S. aureus*, and *Mycoplasma* spp., reside in the udders and, during milking, spread from infected to healthy udders through milking equipment, farm workers’ hands, or other forms of cross-contamination [9,31].

Mastitis-causing pathogens vary geographically: contagious pathogens, such as *S. aureus* and *S. agalactiae*, dominate in Europe and North America, whereas environmental pathogens, such as *E. coli*, *S. uberis*, and *Klebsiella* spp., are more prevalent in Asia and Africa, while Latin America and Oceania show a mix of both types [32,33,34]. This geographical variation mainly arises from differences in hygienic practices in dairy cattle, such as herd management, housing hygiene, and milking practices.

Studies show that contagious and environmental pathogens differ in their resistance patterns. Contagious pathogens, including *S. aureus*, often show resistance to beta-lactams, tetracyclines, and methicillin (MRSA), whereas environmental pathogens, including *E. coli* and *Klebsiella* spp., commonly exhibit resistance to penicillin and tetracycline [29,35,36,37]. Contagious pathogens mainly spread between cows and acquire resistance primarily in response to therapeutic treatments, while environmental pathogens are exposed to diverse antimicrobial pressures in farm environments, promoting multidrug resistance [38,39,40].

The risk of mastitis is further influenced by host-related factors, such as the cow’s age, breed, production level, lactation number, and immune status, as well as management practices [4,23]. Older cows, those with poor immune responses, and certain genetically predisposed breeds (e.g., Holstein-Friesian) are more susceptible to infection [41]. Additionally, suboptimal milking hygiene, inadequate equipment sanitation, and stressful housing conditions exacerbate the risk of infection [42]. Furthermore, poor nutritional management, high-yielding cattle, high milking frequency, and poor udder care practices also increase the prevalence of bovine mastitis [43].

## 3. Antimicrobials Used to Treat Bovine Mastitis

Dairy farms frequently use antimicrobials to prevent and treat mastitis and other diseases, such as pneumonia, diarrhea, lameness, and metritis [44]. Notably, approximately 60–70% of antimicrobial use in these farms is for treating and preventing mastitis [14,45]. Antimicrobials are used in bovine mastitis primarily for therapeutic, prophylactic, and metaphylactic purposes [46]. The choice of antimicrobial depends on factors such as the causative pathogen, its susceptibility profile, the severity of the infection, and the stage of lactation. Beta-lactams (e.g., penicillins, cephalosporins) are commonly used for mastitis caused by Gram-positive bacteria such as *Staphylococcus* spp. and *Streptococcus* spp., whereas aminoglycosides and tetracyclines may be recommended for Gram-negative bacteria (*E. coli*) [47,48]. Dry-cow therapy is used to eliminate existing intramammary infections (IMIs) and to prevent new infections during the dry period. Gentamycin, ampicillin, penicillin, tetracycline, ceftiofur, erythromycin, streptomycin, and fluoroquinolones, administered via intravenous injections or intramammary infusion, are common antimicrobials used worldwide to treat and prevent mastitis caused by IMIs [49,50]. Antimicrobial use for bovine mastitis varies regionally due to differences in regulations and veterinary practices, which causes variations in resistance patterns in pathogens. Europe and North America primarily use beta-lactams, cephalosporins, and macrolides under strict prescription rules, while Asia and Africa often rely on broader-spectrum antimicrobials with less oversight, contributing to higher AMR [51,52,53]. Given that mastitis accounts for the majority of antimicrobial use in dairy farms, repeated and often empirical treatment practices impose strong selection pressure that promotes the emergence and persistence of antimicrobial-resistant bacteria, which further survive and proliferate, with downstream risks to human health and environmental dissemination.

## 4. A One Health Perspective

**Figure 1:**
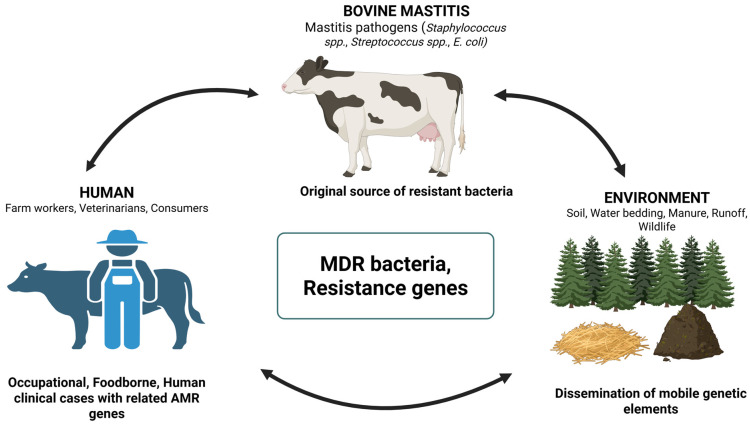
One Health perspective on the emergence and dissemination of bacteria with antimicrobial resistance (AMR) and resistance genes in bovine mastitis, illustrating the interconnected risks across animals, humans, and the environment. All arrows indicate potential routes of pathogen or resistance gene transmission.

AMR associated with bovine mastitis represents a complex challenge that extends beyond animals. The One Health perspective recognizes that resistant pathogens and antimicrobial resistance genes can circulate among dairy cattle, humans, and the environment through interconnected pathways (Figure 1). In the context of bovine mastitis, the broader AMR ecology is the collective result of antimicrobial use in dairy herds to prevent and manage bovine mastitis; exposure of farm workers, veterinarians, and consumers; and environmental dissemination through manure, wastewater, and farm runoff [54,55,56]. Evidence of One Health associations between AMR bacteria isolated from cows with bovine mastitis and animal, human, and environmental domains is summarized in Table 1. Direct animal-to-animal contact and shared milking equipment facilitate the spread of resistant bacteria within the herd [57,58]. Similarly, zoonotic transmission to humans occurs through various mechanisms: farm workers may be exposed during milking and milk handling, or through contact with contaminated farm surfaces; veterinarians while treating animals; and consumers when eating dairy or meat products [46]. Environmental reservoirs, including contaminated bedding, wastewater, and manure, serve as persistent sources of resistant pathogens [59,60]. Cows excrete resistant bacteria and antimicrobial residues into manure and wastewater, which can contaminate the water, soil, and farm environment, facilitating environmental dissemination. Adopting a One Health perspective therefore provides a comprehensive approach for understanding the emergence and potential transmission of AMR associated with bovine mastitis across the animal, human, and environmental domains.

### 4.1. Antimicrobial Resistance in Bovine Mastitis Pathogens

AMR in bovine mastitis pathogens is a serious issue globally [28]. Studies in Ethiopia reported that at least 40–74% of cows in a herd are affected by mastitis [65,66]. Therefore, farmers heavily rely on antimicrobials to prevent and treat mastitis. For instance, one study showed that about 85% of cows in the USA had been treated with at least one antimicrobial for the prevention or treatment of mastitis [67]. The use of antimicrobials to treat mastitis varies; one study showed that 67% of cows on 30 dairy farms received medication for mastitis over a year [68], while another revealed that nearly all cows (93.0%) were treated with dry-cow intramammary antimicrobials at dry-off [69]. Antimicrobial use, especially prolonged administration or broad dry-cow therapy, creates selective pressure that allows resistant bacteria to persist in the udder or environment and facilitates the transfer of resistance genes to other bacteria [70]. In consequence, these factors create higher antimicrobial selection pressure on pathogens and result in the development of resistance. Furthermore, higher doses or multiple antimicrobials become increasingly necessary, eventually exacerbating the severity of infections [71]. A meta-analysis of antimicrobial resistance patterns in *S. aureus* isolated from bovine mastitis in cattle worldwide (1969–2020) shows an increasing trend over time, becoming more apparent from 2009 onwards [72]. Furthermore, this meta-analysis shows that the most prevalent resistance type in *S. aureus* was resistance to penicillin, followed by clindamycin, erythromycin, and gentamicin. Studies have demonstrated that, globally, bacterial species that cause bovine mastitis exhibit resistance to multiple antimicrobials [73,74,75] (Table 2). As a consequence, these bacteria become reservoirs of resistance; the transfer of resistant bacteria and mobile genetic elements to other livestock and humans, either directly or through food products, has become a serious public health threat [76]. The persistence of antimicrobial residues in milk, dairy products, and meat contributes to resistance in the human gut microbiota, reducing the efficacy of antimicrobials; however, this risk is minimized in developed countries with strong regulatory oversight, though exposure can still occur indirectly through milk from cows with subclinical mastitis, environmental contamination, and pathways such as manure and wastewater [77]. Therefore, the seriousness of AMR in bovine mastitis pathogens underscores the urgency of deploying integrated strategies across veterinary, human, and environmental health domains.

### 4.2. Zoonotic and Public Health Implications

AMR in bovine mastitis pathogens has significant implications in zoonotic and public health, posing a serious threat to both animal and human health [89]. Resistant pathogens associated with mastitis, such as *S. aureus*, *S. agalactiae*, *E. coli*, *M. bovis*, *C. bovis*, *Mycoplasma* spp., and *Klebsiella* spp., can transfer from livestock to humans through direct contact or via contaminated raw milk, other dairy products, and meat—particularly in regions with limited pasteurization or post-processing decontamination practices—leading to infections that are increasingly difficult to treat [90,91]. For example, one study showed that MRSA isolated from cows with bovine mastitis and from exposed humans had identical antimicrobial susceptibility profiles and indistinguishable genotypes (ST398, spa type t034), providing direct evidence of MRSA transmission between cows and humans [54]. These transmissions increase the risks of foodborne illness, infection with resistant pathogens, and antimicrobial resistance issues. This zoonotic potential is compounded by the presence of mobile genetic elements, such as plasmids and transposons, which facilitate the horizontal transfer of resistance genes between bacteria in animals and humans [92]. These shared resistance mechanisms bridge the boundaries between veterinary and human medicine, contributing to the global AMR crisis. Additionally, farm environments act as reservoirs for resistant bacteria, with manure, water, and soil providing pathways for resistance to spread beyond the farm environment into broader ecosystems [93]. This amplifies the risk of human exposure to resistant pathogens, particularly for individuals working closely with livestock or consuming improperly processed dairy products. Therefore, the zoonotic and public health implications of AMR in bovine mastitis pathogens highlight the urgent need for a One Health approach that integrates antimicrobial stewardship, robust food safety measures, and surveillance systems across the human, animal, and environmental health domains to mitigate the cascading risks of AMR.

### 4.3. Environmental Reservoirs of AMR

Resistant bacteria originating from mastitis-affected cattle enter the environment through manure, milk, and other waste products, contaminating soil, water, and animal housing facilities [94]. The sources of antimicrobial resistance genes (ARGs) from environment-related bovine mastitis are summarized in Table 3. Environmental pathogens, such as *S. uberis*, *S. dysgalactiae*, coliforms, and *T. pyogenes*, originate from the cow’s environment [10,95]. One study showed that dairy farm waste was a source of 14 beta-lactam resistance genes, including *TEM-1*, *CTX-M-55*, *EC-15*, *CTX-M-14*, *ampC*, and *CTX-M-65*, and 5 multidrug resistance genes, including *soxS*, *soxR*, and *marA* [96]. Dairy farm environments, workers, and water sources were also shown to be sources of AMR [97,98]. These contaminated environments act as breeding grounds for resistant pathogens and serve as hotspots for the exchange of resistance genes via mobile genetic elements such as plasmids and transposons [99]. Eventually, resistant pathogens and genetic materials persist in the environment and re-enter livestock populations or human food chains. Table 1 demonstrates how the environment can play a significant role as a reservoir in disseminating resistant pathogens that cause bovine mastitis. Pathogens associated with bovine mastitis are frequently isolated from farm equipment, bedding materials, manure, and soil surrounding the farms [40,54,64]. Addressing the environmental dimension of AMR in bovine mastitis pathogens necessitates comprehensive strategies, including proper waste management, reduced antimicrobial use, and robust biosecurity measures to break the cycle of resistance dissemination. A One Health approach that integrates environmental stewardship with animal and human health interventions is essential for mitigating the risks associated with these environmental reservoirs of resistance. These environmental reservoirs act as hidden nodes in the One Health network, perpetuating AMR transmission back to livestock and ultimately to humans.

## 5. Integrated One Health Policies to Mitigate AMR in Bovine Mastitis Pathogens

**Figure 2:**
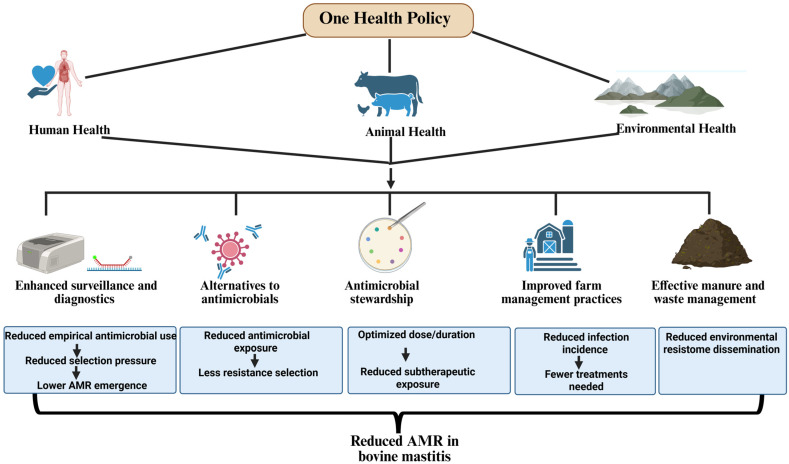
Integration of One Health strategies to mitigate antimicrobial resistance (AMR) in bovine mastitis, along with their biological or epidemiological mechanisms.

AMR in bovine mastitis pathogens represents a significant challenge at the animal, human, and environmental interface, emphasizing the need for a One Health approach with coordinated actions, such as enhanced surveillance and diagnostics, alternatives to antimicrobials, improved farm management practices, and effective manure and waste management, as shown in Figure 2. This figure demonstrates how coordinated measures can reduce antimicrobial use, limit the emergence of resistant bacteria, and prevent dissemination across the farm environment and to humans. The multidisciplinary approach enhances livestock productivity, safeguards public health, and limits environmental dissemination of resistance genes, ensuring a sustainable future for agriculture and healthcare.

Integrating One Health principles into AMR mitigation strategies involves tackling key risk factors associated with bovine mastitis. Poor farm management practices, including inadequate hygiene during milking, improper housing, and reliance on antimicrobials as a first line of defence, significantly contribute to the emergence of resistant pathogens. The One Health approach promotes improvements in these practices through biosecurity measures, better milking hygiene, and sustainable waste management [104]. For example, manure, a recognized reservoir for resistance genes, must be managed using technologies like composting and anaerobic digestion to reduce microbial loads and the potential for environmental dissemination of AMR [105].

### 5.1. Enhanced Surveillance and Diagnostics

Enhanced surveillance and diagnostics are fundamental to mitigating AMR in bovine mastitis pathogens [37,106]. Implementing robust monitoring systems that track antimicrobial use and resistance patterns in mastitis pathogens provides critical insights into the dynamics of resistance development, guiding interventions at both the farm and policy levels [37]. By using pathogen-specific diagnostics, farmers and veterinarians can ensure that antimicrobials are prescribed only when necessary and that treatments are tailored to the specific pathogen responsible for the infection. This targeted approach not only minimizes the use of broad-spectrum antimicrobials but also helps reduce the selection pressure on bacteria, thus slowing the spread of resistance [107]. Additionally, utilizing advanced molecular tools for the early detection of resistance genes in bacteria facilitates the rapid identification of resistant strains, enabling timely and effective treatment strategies and limiting the persistence and transmission of resistant bacteria [108]. Together, these diagnostic innovations preserve antimicrobial efficacy while reducing AMR risks relevant to animal, environmental, and public health.

### 5.2. Alternatives to Antimicrobials

Plant-derived and other potential alternatives used to treat bovine mastitis are summarized in Table 4. This table summarizes reported herbal, botanical, and non-antimicrobial interventions for preventing and treating bovine mastitis. Alternatives to antimicrobials are critical in reducing reliance on antimicrobials and combating AMR in bovine mastitis pathogens [14,54]. *Leptospermum scoparium* and *Origanum vulgare* improved the physical condition of the udder and decreased the somatic cell count (SCC) and white blood cells (WBCs) in cows affected with subclinical mastitis, with strong antimicrobial activities against *Staphylococcus* spp., *S. aureus*, and *E. coli* [109,110]. The use of probiotics, prebiotics, and immunomodulators is a promising strategy to enhance the innate immunity of cattle, thereby reducing the susceptibility to infections and the need for antimicrobial interventions [111,112]. By promoting a healthy gut microbiome and stimulating the immune system, these alternatives can help prevent and control mastitis without the drawbacks of antimicrobial use. Additionally, the development and application of vaccines targeting common mastitis pathogens offer a proactive approach to reducing infection rates, ultimately lowering the need for therapeutic antimicrobials [113]. Other emerging approaches being explored are bacteriophage therapy and antimicrobial peptides, which represent novel, non-traditional treatments for mastitis-causing bacteria [114]. Bacteriophages are naturally occurring viruses that target specific bacterial pathogens; however, their use for mastitis treatment remains in the experimental stage and faces regulatory challenges related to approval processes and clinical implementation. Antimicrobial peptides possess broad-spectrum antimicrobial activity and hold the potential to complement or replace conventional antimicrobials, further reducing the selection pressure for resistant strains. These alternative approaches are essential in establishing sustainable, antimicrobial-free solutions for mastitis management, minimizing the risk of AMR while improving animal health and welfare. However, the application of these alternatives is hindered by multiple factors, such as limited vivo efficacy, regulatory challenges, high costs, and a lack of long-term effectiveness data, emphasizing the need for further studies prior to large-scale implementation.

### 5.3. Antimicrobial Stewardship

The antimicrobial stewardship approach involves the efficient use of antimicrobials, aiming to reduce their misuse and overuse [135]. This is crucial in combating AMR in bovine mastitis pathogens by ensuring the responsible and judicious use of antimicrobials in veterinary practice [136]. Antimicrobial stewardship principles include optimized dosing, selective treatment, and reduced blanket antimicrobial use (e.g., administration of antimicrobials to all animals), minimizing subtherapeutic exposure and preventing enrichment of resistant bacteria in mastitis-affected herds [137]. Establishing clear guidelines for antimicrobial use, including restrictions on prophylactic and growth-promoting applications, is vital to reducing unnecessary antimicrobial exposure and minimizing the risk of resistance development [135]. These guidelines should prioritize treatment based on pathogen-specific diagnostics, ensuring that antimicrobials are only used when necessary and that they target the specific infection. In addition, providing education and training programmes for veterinarians and farmers on the principles of responsible antimicrobial use is essential for fostering a culture of stewardship within the farming community [138]. Such initiatives should focus on understanding the consequences of misuse, promoting alternatives to antimicrobials, and encouraging preventive practices to reduce infection rates. Furthermore, promoting the practice of record-keeping for antimicrobial treatments enhances accountability and traceability, allowing for better monitoring of antimicrobial use and facilitating more informed decision-making in dairy farms [139]. By improving transparency and oversight, antimicrobial stewardship ensures that antimicrobials remain effective in the long term, safeguarding both animal and human health and delaying or mitigating the AMR [140].

### 5.4. Improved Farm Management Practices

Improved farm management practices are essential for preventing and controlling bovine mastitis while minimizing the need for antimicrobial application. Adopting best practices for milking hygiene, such as proper udder preparation, use of clean and sanitized milking equipment, and post-milking teat disinfection, is crucial in reducing the risk of pathogen introduction and transmission [141]. These practices help maintain the health of the udder, lowering the incidence of mastitis and the subsequent reliance on antimicrobials. Additionally, improving housing conditions by ensuring clean, dry bedding and adequate ventilation is vital for minimizing environmental contamination and reducing pathogen load in the barn [142]. Poor housing conditions can contribute to stress and infection, increasing the susceptibility of cattle to mastitis. Implementing robust biosecurity measures, including isolating new or sick animals and controlling the movement of personnel and equipment, prevents the introduction and spread of infectious pathogens within and between farms [143]. By focusing on these proactive, preventive measures, farms can significantly reduce infection rates, promoting animal welfare and decreasing the need for antimicrobials, thereby helping to combat antimicrobial resistance in bovine mastitis pathogens.

### 5.5. Effective Manure and Waste Management

Effective manure and waste management is critical in mitigating the environmental spread of AMR associated with bovine mastitis [144,145]. Farms can help limit the spread of resistance to broader ecosystems by managing manure in ways that prevent the direct runoff of antimicrobials and resistant pathogens into soil and water systems [146]. Composting and anaerobic digestion are effective techniques for reducing the microbial load and resistance genes in manure before field application [147]. These processes not only help break down antimicrobial residues but also lower the number of viable resistant bacteria, making manure safer for agricultural use. As a result of these practices, resistant bacteria are less likely to persist in the environment or be transferred to humans and livestock. Therefore, implementing comprehensive manure and waste management strategies is essential for closing the loop on AMR transmission, promoting sustainable farming practices while safeguarding both animal and public health.

## 6. Conclusions

The One Health perspective offers a holistic solution by addressing AMR at its source while considering its downstream impacts. By fostering cross-sector collaboration, promoting sustainable farming practices, and leveraging scientific innovations, the One Health framework ensures a balanced approach to managing bovine mastitis, mitigating AMR, and protecting public and environmental health. Such an integrated strategy is vital for preserving the efficacy of antimicrobials, ensuring food safety, and maintaining the health of ecosystems in an increasingly interconnected world.

## Figures and Tables

**Table 1:** One Health association of antimicrobial-resistant (AMR) bacteria isolated from bovine mastitis with animal, human, and environmental domains.

Pathogen	Study Year	Dairy Cows	Human Health	Environmental	Key Findings	References
*Staphylococcus aureus*	2018	Mastitis cases confirmed in multiple dairy cows.	Identical MRSA *S. aureus* strains detected in milker, veterinarian, and household members.	MRSA detected on farm equipment and surfaces, indicating environmental persistence.	First reported MRSA outbreak in Polish dairy cattle; demonstrates on-farm and human transmission, highlighting One Health risks.	[45]
*Staphylococcus aureus*	2002–2004	Common mastitis pathogen with β-lactam resistance; MRSA identified in dairy herds.	Documented evidence of MRSA isolated from both cows with mastitis and farm workers.	MRSA detected in bedding, on milking equipment, and in dust/air on farms.	Phenotypic/genotypic matching indicates probable direct transmission between cows and a human worker.	[61]
*Streptococcus* spp., *Staphylococcus aureus*, *Lactococcus* spp., and *Enterococcus* spp.	2007–2008	Widespread resistance among isolates causing bovine mastitis.	Opportunistic human infections reported; shared resistance genes with human streptococci.	Survives in bedding, manure, soil, and water on dairy farms.	Reduced beta-lactam susceptibility observed in some regions.	[62]
*Streptococcus dysgalactiae*	2020	Different resistance levels.	NA	Either cow-associated or environmentally associated mastitis pathogen; may persist on dairy farms for more than one year.	Shows mixed transmission patterns in dairy herds, with evidence of both contagious spread and environmental persistence across farms.	[63]
*Escherichia coli*, *Salmonella enterica*, and *Staphylococcus aureus*	2024	Emerging mastitis agent with MDR isolates reported. Widespread resistance among isolates.	Resistant isolates can cause severe human infections if transmitted.	Found in bedding, water, and manure; spreads in the farm environment.	Serious threats in mastitis management.	[55,56]
*Escherichia coli*	2019–2025	MDR *E. coli* commonly recovered from mastitis milk; resistance genes.	Presence of similar resistance genes in isolates from dairy workers; potential for foodborne exposure via raw milk.	Detected in manure, farm runoff, and soils; ARG persistence documented on farms.	Genomic resistome/virulome analysis shows shared resistance genes; suggests reservoir potential.	[40,64]
*Enterococcus* spp.	2017–2018	Isolated from mastitis milk samples; species-specific distribution among milk, feces, and milking equipment.	Important opportunistic human pathogens; vancomycin-resistant enterococci are a major public health concern.	Widely distributed in feces, bedding, water, aisles, and milking equipment.	Showed species-specific niche distribution across milk, feces, and farm environments.	[59]
*Enterococcus* spp.	2022–2024	Mastitis isolates often carry *van* resistance genes.	*vanC* genes detected in raw bovine milk, suggesting possible transmission route from dairy to humans.	NA	Evidence of plasmid-mediated sharing of resistance determinants between animal and human isolates.	[57]

NA: Not Available.

**Table 2:** Percentage of resistance to various antimicrobials among different pathogens from cows with bovine mastitis in different countries.

Pathogens	Country	Year	DISC or MIC	Resistance to Different Antimicrobials (%)	References
*Streptococcus uberis*	Ireland	2020	DISC	Erythromycin (15.2%), Pirlimycin (22.2%), Tetracycline (11.5%)	[78]
	Austria	2017	DISC	Penicillin (2.0%)	[79]
*Streptococcus* spp.	Denmark	2016	MIC	Erythromycin (6.6%), Streptomycin (98.4%), Tetracycline (21.3%), Trimethoprim (1.6%)	[80]
	Taiwan	2020–2021	DISC	Tetracycline (86.30%), Neomycin (79.45%), Bacitracin (38.35%), Ampicillin (45.20%), Oxacillin (73.97%), Cefuroxime (19.17%), Cephalothin (8.21%), Ceftiofur (26.02%).	[29]
*Streptococcus agalactiae*	China	2017–2019	DISC	Streptomycin (24.8%), Piperacillin (29.5%), Ceftriaxone (98.1%), penicillin (98.1%), Amoxicillin (98.1%), Ceftazidime (98.1%),	[81]
*Staphylococcus* spp.	Germany	2012	DISC	Penicillin (74.28%), Gentamycin (10%), and Tetracycline (7.14%).	[82]
	Slovakia	2015–2016	DISC	Penicillin (5.9%), Oxacillin (14.4%), Lincomycin (4.8%), Neomycin (20.9%), Streptomycin (36.4%).	[75]
	Taiwan	2020–2021	DISC	Tetracycline (59.37%), Neomycin (21.87%), Bacitracin (34.37%), Ampicillin (43.75%), Oxacillin (53.12%),	[29]
*Staphylococcus aureus*	India	2021	DISC	Penicillin (83.64%), Cefuroxime (21.82%), Amikacin (58.18%), Gentamicin (34.55%), Oxytetracycline (98.18%), Lincomycin (49.09%)	[83]
	Kenya	2018–2019	DISC	Ampicillin (71.4%), Streptomycin (21%), Gentamycin (6%), Ciprofloxacin (3.2%), Norfloxacin (4.3%), Tetracycline (21%), Erythromycin (25.2%), Chloramphenicol (8.7%)	[84]
Coliforms	Taiwan	2020–2021	DISC	Tetracycline (31.57%), Neomycin (21.05%), Bacitracin (68.42%), Ampicillin (31.57%), Oxacillin (100%), Cefuroxime (15.78%), Cephalothin (31.57%)	[29]
*Escherichia coli*	Finland	2011	MIC	Ampicillin (18.7%), Chloramphenicol (6.9%), Kanamycin (6.3%), Streptomycin (18.1%), Tetracycline (16.7%), Sulfamethoxazole (14.6%), Trimethoprim (10.4%)	[85]
	Germany	2017	MIC	Ampicillin (12.1%), Ceftiofur (4.5%), Tetracycline (8.5%), Gentamicin (0.9%), Ciprofloxacin (2.2%)	[86]
*Klebsiella pneumoniae*	Denmark	2016	MIC	Ampicillin (83.3%), Streptomycin (5.6%)	[80]
	China	2019	MIC	Amoxicillin (100%), Clavulanate (100%), Cefquinome (30.0%), Polymyxin B (30%), Tetracycline (30%), Kanamycin (30%), Ceftiofur (20%)	[87]
	Sweden	2013	MIC	Ampicillin (95.4%), Colistin (4.6%), Ciprofloxacin (4.6%), Tetracycline (9.1%)	[88]

DISC: disc diffusion; MIC: minimum inhibitory concentration.

**Table 3:** Sources of antimicrobial resistance genes (ARGs) and virulence genes in dairy farms with potential to cause mastitis.

Pathogens	Antimicrobials	Source	ARGs	Virulence Genes	Major Findings	References
*Escherichia coli*	Aminoglycosides and beta-lactams	Dairy farm waste	14 beta-lactam resistance genes, including *TEM-1*, *CTX-M-55*, *EC-15*, *CTX-M-14*, and *ampC*; 5 multidrug resistance genes, including *soxS*, *soxR*, *AcrAB-TolC-MarR*, and *marA*	40 different adherence-related virulence factors, including *ecpA*, *elfA*, *eaeH*, *hcpA*, *fimA*, *fimG*, and *fimI*	48.4–100% isolates exhibited resistance to the tested antimicrobials	[96]
*Escherichia coli*	18 antimicrobials, including ampicillin and carbenicillin	Water source in a dairy farm	*blaTEM*, *blaCMY-2*, *blaSHY*, *aac(3)IIa*, and *aadA*	NA	Resistance to ampicillin and carbenicillin was the most common Strong potential of *E. coli* to transfer ARGs to other pathogens	[100]
*Staphylococcus* spp.	Erythromycin, oxacillin, cephalothin, and gentamicin	Dairy farm environment	*Bap*, *icaA*, and *mecA*	NA	Mainly resistant to erythromycin (23%) and oxacillin (16%)	[101]
*Staphylococcus* spp.	15 antimicrobials, including amoxicillin, ampicillin, and cefoxitin	Humans working with dairy animals	*mecA*	NA	Multidrug resistance was common	[102]
*Staphylococcus* spp.	13 antimicrobials, including beta-lactams	Milker’s hands, liners, calves	*mecA*	*sea*, *see*, *eno*, *can*, *ebps*, *fnbA*, and *coa*	Most of the isolates were resistant to tested antimicrobials	[103]
*Staphylococcus* spp.	Beta-lactams, cephalosporins, tetracycline, ciprofloxacin, and gentamicin	Milking parlour, workers’ nasal cavities	*blaZ*, *aacA-aphD*, *ermC*, *tetK*, and *mecA*	NA	Prevalence of AMR *Staphylococcus* was high in milking parlour environmental samples	[98]
NA	Penicillins	Bovine feces	*blaTEM*	NA	Dairy farms could be considered a hotspot of antimicrobial ARGs	[97]

ARGs: antimicrobial resistance genes; NA: Not Available.

**Table 4:** Plant-derived and other alternatives used to treat bovine mastitis.

Alternatives	Major Findings	References
*Leptospermum scoparium* and *Origanum vulgare*	Antimicrobial activity against *Staphylococcal* and *E. coli*.	[110]
*Oregano vulgare*	Improves the physical condition of the udder and decreases SCC and WBC in cows affected with subclinical mastitis. Prevents the growth of *S. aureus* and *E. coli*.	[109]
*Citrus × sinensis*	Prevents *S. aureus* growth and biofilm formation, and reduces adhesion and invasion.	[115]
*Minthostachys verticillate*	Antibacterial capacity and anti-biofilm effect against *E. coli*, *Bacillus pumilus*, and *Enterococcus faecium*.	[116]
*Thymus vulgaris*, *Oregano vulgare*, *Origanum majerana*	Reduce the growth of *Prototheca zopfii* with resistance to fluconazole and flucytosine.	[117]
*Alpinia purpurata*	Bactericidal effects on *S. epidermidis*, *S. aureus*, and *S. agalactiae*. Curcumin and gingerol killed bacteria by disrupting their extracellular membrane.	[118]
*Taraxacum officinale*	Free radical scavenging, antioxidant, antibacterial, and anti-inflammatory activities. Downregulates the inflammatory response.	[119,120]
Nisin	Produced by *Lactococcus lactis*; showed antimicrobial activity against Gram-positive bacteria.	[121]
Polybia MP-1	A 14-amino acid peptide from wasp venom with bactericidal activity against multidrug-resistant *S. aureus*, *E. coli*, and *Klebsiella pneumoniae*.	[122,123]
Lactoferrin	A multifunctional glycoprotein found in saliva, tears, bronchial mucus, colostrum, and milk, with antimicrobial, anti-inflammatory, immunomodulatory, anticatabolic, and antioxidative effects.	[124,125]
Bacteriophages	Target and lyse mastitis-causing bacteria, such as *S. aureus*, *E. coli*, and *S. uberis*, by injecting their genetic material into bacterial cells, replicating inside the bacteria, and causing cell lysis.	[14,126]
Vaccination	Stimulates the immune system to recognize and respond to bacteria. Enhances adaptive immunity, promoting antibody production and immune memory. Boosts the neutrophil response, improving bacterial clearance and reducing inflammation. Toxoids in the vaccine neutralize bacterial toxins and adhesion inhibitors to prevent bacterial colonization.	[127,128]
Probiotics	Feeding probiotics to heifers and transition cows reduced the incidence of clinical mastitis, lowered SCC, and minimized days of discarded milk. Supplementation with Lactobacilli, yeast, and a lactic acid bacterium–maltodextrin mixture optimized the mammary microbiota and enhanced mammary resistance in dairy cows.	[129,130]
Stem cells	Intramammary administration of adipose tissue-derived mesenchymal stem cells (AT-MSCs) eliminated *S. aureus* in the udder. MSCs exhibit immunomodulatory properties by secreting bioactive compounds and facilitating the repair of damaged tissues.	[131,132]
Nanotechnology-based therapy	A self-assembling tilmicosin nanogel had a higher cure rate against *S. aureus*-infected mastitis cows compared to conventional treatment methods. Cinnamon oil and silver nanoparticles exhibited bactericidal activity against *S. agalactiae*.	[133,134]

## Data Availability

No new data were created or analyzed in this study. Data sharing is not applicable to this article.
